# Haemodynamic consequences of changing potassium concentrations in haemodialysis fluids

**DOI:** 10.1186/1471-2369-12-14

**Published:** 2011-04-06

**Authors:** Luca Gabutti, Igor Salvadé, Barbara Lucchini, Davide Soldini, Michel Burnier

**Affiliations:** 1 Division of Nephrology, Ospedale la Carità, Via Ospedale, 6600 Locarno, Switzerland; 2 Department of Internal Medicine, Ospedale la Carità, Locarno, Switzerland; 3 Division of Nephrology, University Hospital of Lausanne, Lausanne, Switzerland

**Keywords:** Haemodynamics, hypotension, potassium, haemodialysis, dialysis fluids

## Abstract

**Background:**

A rapid decrease of serum potassium concentrations during haemodialysis produces a significant increase in blood pressure parameters at the end of the session, even if effects on intra-dialysis pressure are not seen. Paradoxically, in animal models potassium is a vasodilator and decreases myocardial contractility. The purpose of this trial is to study the precise haemodynamic consequences induced by acute changes in potassium concentration during haemodialysis.

**Methods:**

In 24 patients, 288 dialysis sessions, using a randomised single blind crossover design, we compared six dialysate sequences with different potassium profiles. The dialysis sessions were divided into 3 tertiles, casually modulating potassium concentration in the dialysate between the value normally used K and the two cut-off points K+1 and K-1 mmol/l. Haemodynamics were evaluated in a non-invasive manner using a finger beat-to-beat monitor.

**Results:**

Comparing K-1 and K+1, differences were found within the tertiles regarding systolic (+5.3, +6.6, +2.3 mmHg, p < 0.05, < 0.05, ns) and mean blood pressure (+4.3, +6.4, -0.5 mmHg, p < 0.01, < 0.01, ns), as well as peripheral resistance (+212, +253, -4 dyne.sec.cm^-5^, p < 0.05, < 0.05, ns). The stroke volume showed a non-statistically-significant inverse trend (-3.1, -5.2, -0.2 ml). 18 hypotension episodes were recorded during the course of the study. 72% with K-1, 11% with K and 17% with K+1 (p < 0.01 for comparison K-1 vs. K and K-1 vs. K+1).

**Conclusions:**

A rapid decrease in the concentration of serum potassium during the initial stage of the dialysis-obtained by reducing the concentration of potassium in the dialysate-translated into a decrease of systolic and mean blood pressure mediated by a decrease in peripheral resistance. The risk of intra-dialysis hypotension inversely correlates to the potassium concentration in the dialysate.

**Trial Registration Number:**

NCT01224314

## Background

Kidneys are responsible for long-term potassium homeostasis; this exposes patients with end-stage renal disease to a high risk of hyperkalaemia [1-5]. Recovering potassium homeostasis is one of the important objective of dialysis. Considering that its location is mainly intracellular (98% of the pool [1]), its potential removability during a haemodialysis session is quantitatively modest (between 40 and 80 mmol corresponding to 1-2% of total body potassium) [6]. As a consequence, even if, in order to be suitable, potassium removal during dialysis should be equal to the amount accumulated during the inter-dialytic phase, in clinical practice the potassium concentration in the dialysate is usually adjusted with the suboptimal goal of avoiding pre-dialysis hyperkalaemia [7].

The importance of the body content and serum concentration of potassium to control blood pressure remains controversial. Epidemiological data suggest a role for potassium depletion as a co-factor in the development and severity of hypertension, while dietary potassium inversely correlates with blood pressure [8-10]. In animal models, an acute decrease in serum potassium concentration produces vasocostriction mediated by the vascular endothelium and an increase in myocardial contractility; the opposite effect is observed if it increases [11,13].

In haemodialysis nephrologists are faced with sudden changes in blood pressure and haemodynamic fragility phases that have a multi-factorial origin; ultrafiltration, decrease in osmolarity with imbalance and correction of metabolic acidosis play a predominant role [7,14-19]. Despite this, and thanks to some strategies based on current practice, with particular reference to calcium and magnesium concentration in the dialysate [16,20], dialysate temperature [21] and ultrafiltration and sodium concentration profiles [7,22-25], pressure stability is guaranteed as a general rule. Some electrolytes, particularly sodium and bicarbonate, can be modulated in profiles with the purpose of better respecting the gap in osmolarity or concentration that is established during the haemodialysis session, but their haemodynamic effect still remains controversial [21,23,25].

Serum potassium is an electrolyte whose concentration - in order to guarantee a negative balance - varies rapidly and significantly during dialysis, frequently resulting in going from pre-dialysis hyperpotassaemia to intra-dialysis hypopotassaemia. In a study performed by Dolson, designed to analyze the consequences of acute potassium changes on haemodynamics, differences in intra-dialytic blood pressure were not found between the groups treated with dialysates containing 1, 2 or 3 mmol/l of potassium [6]. However, at the end of the dialysis session those patients treated with the lower potassium concentrations showed what was called a "rebound hypertension" [6].

With the purpose of better characterising this phenomenon, we redesigned the study dividing the dialysis session into 3 phases (in fact, clinical practice suggests that the haemodynamic pattern at the beginning, intermediate and final phases of the dialysis are not the same) and programming for each a more or less sharp drop in serum potassium concentration, respecting in the meantime the need to remove the amount of potassium that usually keeps the patient in steady-state. Using a crossover protocol, we divided the dialysis session into 3 tertiles where the potassium concentration in the dialysate was modulated between the usual concentration for the study subject and two cut-off points at +1 e -1 mmol/l respectively. To complete the information provided by blood pressure, haemodynamics were measured in a non-invasive manner using a finger beat-to-beat monitor.

The primary end point was the difference in haemodynamic parameters between the extremes in potassium concentration of the dialysate, while the incidence of hypotension during dialysis was considered a secondary end point.

## Methods

Twenty-four chronic haemodialysis patients (13 male and 11 female) were enrolled in the study. Each patient was dialysed for 3 to 4 hours and 30 minutes three times a week and was clinically stable and without intercurrent illnesses. Using a single blind crossover design, patients were randomised in the six dialysate potassium sequences of the study. Each dialysis session was divided into three equal parts (tertiles): during one part the potassium concentration of the dialysate was the same as the one usually prescribed to the patient, whereas during the other two parts it was either increased or reduced by 1 mmol/L. The 6 different permutations were repeated twice, so that each patient underwent 12 dialysis sessions during the study (see Table 1 for sequence details).

**Table 1:** Dialysate potassium sequences.

Sequence	Dialysate Potassium		
	1st tertile	2nd tertile	3th tertile
**1**	K-1	K	K+1
**2**	K-1	K+1	K
**3**	K	K-1	K+1
**4**	K	K+1	K-1
**5**	K+1	K	K-1
**6**	K+1	K-1	K

Dialysate potassium sequences used in the study. The population was randomised to begin with one of the six sequences. (K = usual potassium concentration (range 2 to 4 mmol/l), K+1 and K-1 = + or -1 mmol/L of potassium)

The haemodialyses were performed using a 4008 H machine, equipped with a cartridge of bicarbonate Bibag^©^, and a high flux single use polysulfone membrane, all from Fresenius Medical Care (Bad Homburg, Germany). The prescribed dialyser effective surface area, dialysis fluid conductibility, dialysate temperature and composition (with the exception of potassium concentration), effective blood flow, and dry weight were recorded at the enrolment in the study and were then left unchanged. The medications of the patients were also left unchanged. Serum potassium and patient weight were measured at the beginning and at the end of each dialysis session. Blood samples were taken from the arterial limb of the shunt.

Kt/V was used to quantify haemodialysis adequacy and was calculated using a second generation single-pool Daugirdas formula (Kt/V = -ln(R-0.03) + [(4-3.5 × R) × (UF/W)], where R = post-dialysis BUN/pre-dialysis BUN, UF = net ultrafiltration, W = weight, K = dialyzer clearance of urea, t = dialysis time, and V = patient's total body water.

The incidence of hypotension episodes (defined as a systolic blood pressure <90 mmHg) was recorded.

Systolic and diastolic blood pressures, heart rate, stroke volumes (integrated mean of the flow waveform between the current upstroke and the dicrotic notch) and total peripheral resistances (ratio of mean arterial pressure to stroke volume multiplied by heart rate) were evaluated at the beginning of the session and then every 30 minutes using a Finometer^© ^finger beat-to-beat monitor (Finapres Medical Systems BV, Arnhem, The Netherlands). Finometer^© ^measures finger blood pressure noninvasively on a beat-to-beat basis and gives waveform measurements similar to intra-arterial recordings.

Mean blood pressure (BP_mean_) was calculated using the following formula: BP_mean _= (BP_syst_+2BP_dias_)/3, where BP_syst _and BP_dias _are systolic and diastolic blood pressure, respectively.

The fluid loss as a function of the time was considered to be constant during the dialysis session and was recorded as total ultrafiltration. The dry weight was established on the basis of clinical assessment and bioimpedance (Body Composition Monitor, Fresenius Medical Care; Bad Homburg, Germany).

Statistical analyses were performed using the SAS System (Statistical Analysis System). Comparisons between body weight, potassium concentration and haemodynamic parameters were done first with an ANOVA followed, if significant by a paired t-test performed between the mean values obtained in each patient with each modality. To improve the probability of showing significant differences, the haemodynamic parameters within the tertiles were compared against the dialysate potassium concentration cut-off points (-1 vs. +1 mmol/l). Percentages were compared using a Fisher Exact test. In all cases, a *P *≤ 0.05 was considered statistically significant; *P *was expressed as *ns *(not significant) or as significant (*P *≤ 0.05).

The protocol of the study was approved by the local Ethical Committee (Comitato Etico Cantonale del Cantone Ticino). All the patients gave written informed consent prior to enrolling in the study.

## Results

### Characteristics of the studied population

The characteristics of the studied population (*n *= 24) at the moment of enrolment were (mean ± SD): age 70.3 ± 9.8 years, weight 71.8 ± 17.2 kg, male/female ratio 1.18. The basis haemodialyses prescriptions were: dialyser effective surface area 1.79 ± 0.09 m^2^; Kt/V 1.65 ± 0.33; dialysis fluid conductibility 13.8 ms/cm; dialysis fluid temperature 35.5 to 37.5°C; effective blood flow 334 ± 69 ml/min; dialysis fluid flow rate 500 to 800 ml/min, potassium 2 to 4 mmol/l, magnesium 0.5 mml/l, calcium 1.25 to 1.50 mmol/l, acetate 3 mmol/l and glucose 1 g/l (see Table 2 for details including underlying nephropathies, comorbidities, antihypertensive drugs in use, dry weight, dialyser surface area, dialysate duration, and Kt/V).

**Table 2:** Characteristics of the cohort.

Patient number	Sex M/F	Age (y)	Underlying nephropathy	Comorbidities		Medication				Dry weight (kg)	Dialyser surface area(m2)	Dialysis Duration (h)	Kt/V
				Ischemic cardio-myopathy	Diabetes mellitus	Beta-blockers	Calcium antagonists	Alpha-blockers	ACE-inhibitors or ARB				
1	F	75	Diabetic nephropathy	Y	Y	Y	N	Y	Y	72.5	1.8	3.0	1.51
2	F	88	Diabetic nephropathy	N	Y	Y	Y	Y	Y	60.0	1.8	3.0	1.48
3	M	59	Diabetic nephropathy	Y	Y	Y	N	N	Y	100.5	1.8	4.0	1.44
4	M	71	Nephroangiosclerosis	Y	N	Y	N	N	Y	69.5	1.8	4.0	1.66
5	M	59	Focal segmental glomerulosclerosis	N	N	Y	Y	Y	N	79.5	1.8	3.5	1.46
6	M	88	Nephroangiosclerosis	N	N	Y	N	N	N	76.0	1.4	4.0	1.52
7	F	86	Nephroangiosclerosis	Y	N	Y	N	N	Y	43.0	1.8	3.0	1.59
8	M	81	Nephroangiosclerosis	N	N	Y	Y	N	N	79.0	1.8	3.5	1.38
9	F	74	Diabetic nephropathy	N	Y	N	Y	N	N	81.0	1.8	3.5	1.76
10	M	64	Nephroangiosclerosis	N	N	Y	N	N	N	98.0	1.8	3.5	1.41
11	M	74	Nephroangiosclerosis	N	N	N	N	N	N	69.5	1.8	4.0	1.52
12	F	66	Trombotic microangiopathy	N	N	N	Y	N	Y	52.0	1.8	3.5	1.90
13	M	65	Focal segmental glomerulosclerosis	N	N	Y	N	N	N	87.2	1.8	4.0	2.08
14	M	72	Nephroangiosclerosis	Y	N	N	N	N	Y	92.0	1.8	3.0	1.00
15	F	75	Diabetic nephropathy	N	Y	Y	Y	Y	Y	48.0	1.8	3.5	1.56
16	M	77	Proliferative glomerulonephritis	Y	N	Y	N	N	Y	80.5	1.8	3.0	1.07
17	M	72	Diabetic nephropathy	Y	Y	N	N	N	Y	89.5	1.8	4.0	1.37
18	F	65	Diabetic nephropathy	Y	Y	Y	Y	Y	Y	48.0	1.8	3.5	2.08
19	F	56	Nephroangiosclerosis	N	N	Y	Y	N	Y	65.0	1.8	3.5	2.07
20	F	48	IgA nephropathy	N	Y	Y	N	N	N	60.0	1.8	3.5	2.20
21	F	70	Nephroangiosclerosis	N	N	N	Y	N	Y	47.5	1.8	3.5	2.00
22	F	64	Relapsing pyelonephritis	N	N	Y	Y	Y	Y	55.0	1.8	3.3	2.22
23	M	68	IgA nephropathy	Y	N	N	Y	N	N	94.5	1.8	4.0	1.52
24	M	71	Nephroangiosclerosis	Y	Y	Y	N	N	N	75.5	1.8	4.5	1.70

Characteristics of the population at the beginning of the study. (M, male; F, female; Y, yes; N, no; ACE, angiotensin converting enzyme; ARB, angiotensin receptor antagonists)

### Serum potassium and ultrafiltration

The average total ultrafiltration obtained in the 6 sequences of dialysate potassium concentration used for the study did not show any significant differences. Pre-dialysis serum potassium was in turn comparable, while, as expected, post-dialysis serum potassium was influenced mainly by the potassium concentration in the dialysate during the last dialysis tertile, producing significant differences (p < 0.01) between the sequences that ended with the usual dialysate potassium concentration, supplemented by 1 or reduced by 1 mmol/l (Table 3).

**Table 3:** Serum potassium and ultrafiltration.

Sequence	Ultrafiltration (L)	SD	Serum K pre-dialysis (mmol/L)	SD	Serum K post- dialysis (mmol/L)	SD	pre-post dialysis delta K (mmol/L)	SD
**1**	1.68	0.99	4.65	0.77	3.90	0.32	0.83	1.02
**2**	1.61	0.94	4.77	0.66	3.74	0.46	1.03	0.85
**3**	1.54	0.95	4.92	0.71	3.93	0.32	1.05	1.02
**4**	1.64	0.94	4.84	0.84	3.46	0.34	1.38	0.95
**5**	1.51	0.82	4.74	0.69	3.39	0.51	1.31	1.58
**6**	1.64	1.08	4.81	0.70	3.73	0.34	1.11	0.84

Serum Potassium pre- and post-dialysis and mean total ultrafiltration as a function of the dialysate sequences. Between the pairs of sequences 1 and 3, 2 and 6 and 4 and 5, the difference in the mean post-dialysis serum potassium concentration was significant (p < 0.01).

### Effect of the potassium variations on systemic haemodynamics

**Figure 1:**
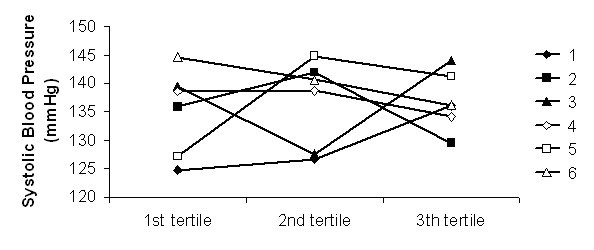
**Systolic blood pressure**. Systolic blood pressure as a function of the dialysis tertiles in the 6 dialysate sequences (1 to 6).

**Figure 2:**
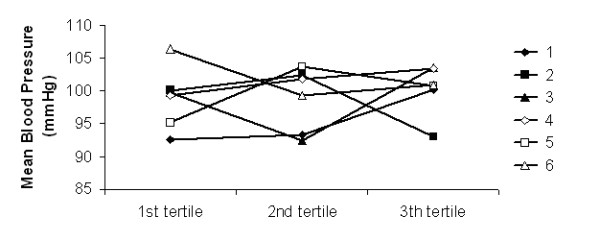
**Mean blood pressure**. Mean blood pressure as a function of the dialysis tertiles in the 6 dialysate sequences (1 to 6).

**Figure 3:**
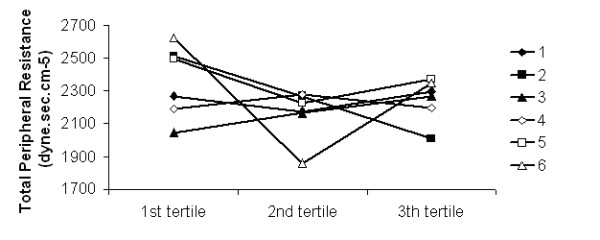
**Peripheral resistance**. Total peripheral resistance as a function of the dialysis tertiles in the 6 dialysate sequences (1 to 6).

**Figure 4:**
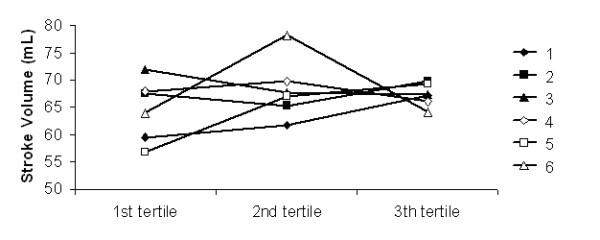
**Stroke volume**. Stroke volume as a function of the dialysis tertiles in the 6 dialysate sequences (1 to 6).

The analysis of the evolution of haemodynamic parameters as a function of dialysis tertiles has not shown any significant differences between the 6 dialysate sequences used during the study (Figures 1, 2, 3 and 4).

If, however, (in post hoc analysis) the mean pressure of the different sequences is taken into account and grouped as a function of dialysate potassium concentration in the first tertile (1 with 2, 3 with 4 and 5 with 6), the differences become significant (p < 0.01). The lowest mean pressure is thus recorded in the sequences that start by inducing the fastest decrease in serum potassium (Table 4).

**Table 4:** Mean haemodynamic parameters.

Sequence	Systolic BP (mmHg)	SD (mmHg)	Mean BP (mmHg)	SD (mmHg)	TPR (dyne.sec.cm-5)	SD (dyne.sec.cm-5)	Stroke volume (mL)	SD (mL)
**1**	129.1	19.9	95.4	12.7	2249	898	62.9	23.0
**2**	135.8	18.0	98.5	10.5	2262	1143	67.5	26.5
**3**	137.0	20.6	98.6	13.6	2160	1134	69.0	23.4
**4**	137.1	19.2	101.5	12.2	2221	1010	67.9	24.3
**5**	137.7	21.2	99.9	12.5	2366	905	64.4	21.0
**6**	140.5	16.4	102.3	12.0	2277	1202	68.7	28.2

Mean values of systolic and mean blood pressure (BP), total peripheral resistance (TPR) and stroke volume for the 3 dialysis tertiles.

**Figure 5:**
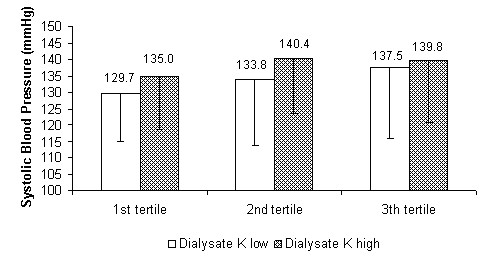
**Systolic blood pressure**. Systolic blood pressure as a function of dialysis tertiles comparing treatments with the two potassium (K) concentration cut-off points in the dialysate (high = K+1 and low = K-1). P for the 1st, 2nd and 3rd tertiles: < 0.05, < 0.05 and ns respectively.

**Figure 6:**
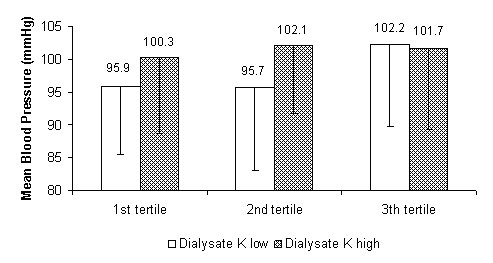
**Mean blood pressure**. Mean blood pressure as a function of dialysis tertiles comparing treatments with the two potassium (K) concentration cut-off points in the dialysate (high = K+1 and low = K-1). P for the 1st, 2nd and 3rd tertiles: < 0.01, < 0.01 and ns respectively.

**Figure 7:**
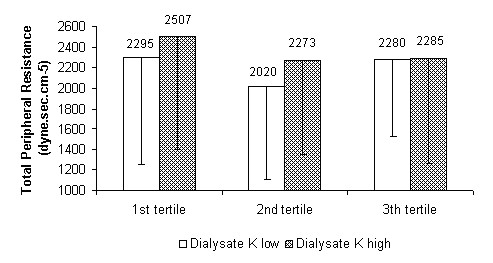
**Total peripheral resistance**. Total peripheral resistance as a function of dialysis tertiles comparing treatments with the two potassium (K) concentration cut-off points in the dialysate (high = K+1 and low = K-1). P for the 1st, 2nd and 3rd tertiles: < 0.05, < 0.05 and ns respectively.

**Figure 8:**
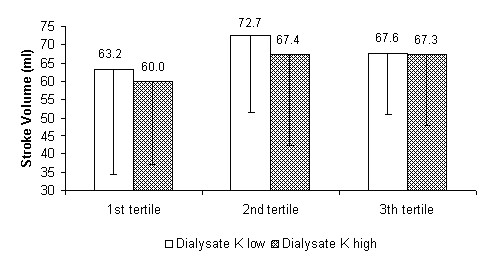
**Stroke volume**. Stroke volume as a function of dialysis tertiles comparing treatments with the two potassium (K) concentration cut-off points in the dialysate (high = K+1 and low = K-1). P for the 1st, 2nd and 3rd tertiles: ns.

At the same time, by comparing - as prespecified - haemodynamic parameters in the treatments performed with the higher and lower potassium concentration in the dialysate, significant differences in systolic and mean blood pressure and peripheral resistance were found within the tertiles. The systolic and mean blood pressure, as well as peripheral resistance, were lower for the first and second tertiles using the dialysate with the lowest potassium concentration. The stroke volume showed a non-statistically-significant inverse trend. In the last tertile the differences were narrowed, losing significance (see Figure 5, 6, 7 and 8 for details).

### Incidence of hypotension episodes

**Figure 9:**
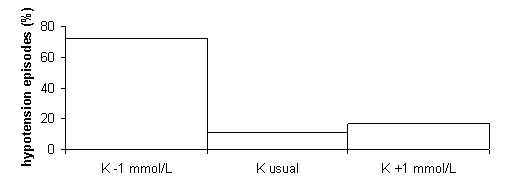
**Incidence of hypotension episodes**. Incidence of hypotension episodes as a function of the dialysate potassium (K) concentration. P for the differences between both K-1 and K and K-1 and K+1 < 0.01; n = 18.

Hypotension episodes were significantly more numerous in the haemodialysis phases where the lower potassium concentrations were used in the dialysate (Figure 9). During the 288 dialysis performed in the study, 18 hypotension episodes (systolic blood pressure <90 mmHg) were recorded, 72% of which occurred in the tertiles with the lower dialysate potassium concentration (11% with the usual concentration and 17% with the higher concentration).

## Discussion

The study showed that a low potassium concentration in the dialysate, inducing a rapid decrease in serum potassium, causes a decrease in systolic and mean blood pressure correlated with a decrease in peripheral resistance. The decrease in pressure recorded when a dialysate with a potassium concentration 1 mmol/l lower than the one usually employed for the patient was used, translated into a higher incidence of hypotension, defined as episodes with systolic blood pressure <90 mmHg (72% of the episodes happened during the tertile using the lowest dialysate potassium concentration). The dialysate potassium concentration in the initial tertile affects mean blood pressure for the whole dialysis session in that a larger gap between serum and dialysate potassium concentrations results in a lower pressure. The effect of removing potassium is progressively reduced during dialysis, with haemodynamic consequences that are no longer significant during the final tertile. The lesser impact during the intermediate and final stages of dialysis could be due to the gap between serum and dialysate potassium, which progressively narrows during the dialysis session.

The data obtained agree with what was observed by Dolson [6] who, while not highlighting pressure discrepancies during the dialysis using dialysates with potassium concentrations of 1, 2 or 3 mmol/l, had observed hypertension, defined as "reflex" (or "rebound"), at the end of the session that used the two lower potassium concentrations. The lack of differences during dialysis in the aforementioned study could have been a consequence of (i) the lower statistical power (11 versus 24 subjects investigated) and (ii) the different method used (based on the steady state prescription, we randomly reduced and increased the dialysate potassium concentration in the whole group). The cited blood pressure rebound after dialysis suggests a counterregulatory phenomenon compatible with an undetected intradialytic hypotension/hypoperfusion phase.

However, if we take into account the experimental data obtained from animals besides the typical metabolic circumstances of kidney failure that requires dialysis, it is surprising to observe the haemodynamic pattern that is traced and which, contrary to expectations, shows a hypertensive effect for acute decrease in serum potassium. In fact, the experimental hypokalaemia model demonstrates a vasoconstriction and an increase in myocardial contractility [11-13]. The difference between the theoretical and the observed pattern could be due to the method employed to evaluate peripheral resistance (indirect, non-invasive measurements using beat-to-beat in our case) and the metabolic circumstances of dialysis with sharp variations in other factors that together can modify haemodynamics (calcaemia, osmolarity, acid-base balance, temperature), as well as concomitant counterregulatory phenomena, particularly the sympathetic and renin-angiotensin systems. The fact that hypokalaemia sensitizes myocardium to hypoxic related dysfunction [26] together with the selection for the study of an elderly population (mean age 70.3 years) with a high incidence of ischemic cardiomyopathy (10 out of 24 subjects) could have influenced the incidence of intra-dialytic hypotensions and the results. Moreover two other reasons could potentially explain a blood pressure reduction related to acute potassium decrease in the dialysis population: hypokalaemia may exacerbate autonomic dysfunction while intra-dialytic potassium loss accounts for a decrease in total osmoles [7].

Regardless of the pathophysiological explanations of the haemodynamic consequences, the results are potentially relevant in that (i) dialysate potassium concentration could theoretically be modulable in a profile, as proposed for other electrolytes like sodium and bicarbonate and (ii) more attention could be paid to controlling the potassium balance with alternative measures (diet, chelating agents, avoidance of medications which inhibits the renin-angiotensin system if unnecessary [27] and possibly prescription of mineralocorticoids [28]).

## Conclusions

In conclusion, a rapid decrease in the concentration of serum potassium during the initial stage of the dialysis - obtained by reducing the concentration of potassium in the dialysate - translates into a decrease of systolic and mean blood pressure mediated by a decrease in peripheral resistance. The risk of intra-dialysis hypotension inversely correlates to the potassium concentration in the dialysate. Based on the results obtained by this study, modulating potassium concentration in the dialysate during haemodyalisis sessions could have favourable haemodynamic consequences.

## Competing interests

The authors declare that they have no competing interests.

## Authors' contributions

LG was involved in the study design, sample collection, analysis and interpretation of the data and in the writing of the report; IS, BL and DS participated in the sample collection, analysis and interpretation of the data and in the writing of the paper, MB helped formulate the study design, the data analysis strategy and contributed to the writing of the paper. All authors have read and approved the final version of the manuscript.

## Pre-publication history

The pre-publication history for this paper can be accessed here:

http://www.biomedcentral.com/1471-2369/12/14/prepub
